# Development and validation of an ensemble classifier for real-time recognition of cow behavior patterns from accelerometer data and location data

**DOI:** 10.1371/journal.pone.0203546

**Published:** 2018-09-07

**Authors:** Jun Wang, Zhitao He, Guoqiang Zheng, Song Gao, Kaixuan Zhao

**Affiliations:** 1 Post-Doctoral Research Station of Control Science and Engineering, Henan University of Science and Technology, Luoyang, Henan, P. R. China; 2 College of Agriculture Equipment Engineering, Henan University of Science and Technology, Luoyang, Henan, P. R. China; 3 College of Information Engineering, Henan University of Science and Technology, Luoyang, Henan, P. R. China; University of Illinois, UNITED STATES

## Abstract

Behaviors are important indicators for assessing the health and well-being of dairy cows. The aim of this study is to develop and validate an ensemble classifier for automatically measuring and distinguishing several behavior patterns of dairy cows from accelerometer data and location data. The ensemble classifier consists of two parts, our new Multi-BP-AdaBoost algorithm and a data fusion method based on D-S evidence theory. We identify seven behavior patterns: feeding, lying, standing, lying down, standing up, normal walking, and active walking. Accuracy, sensitivity, and precision were used to validate classification performance. The Multi-BP-AdaBoost algorithm performed well when identifying lying (92% accuracy, 93% sensitivity, 82% precision), lying down (99%, 82%, 86%), standing up (99%, 74%, 85%), normal walking (97%, 92%, 86%), and active walking (99%, 94%, 89%). Its results were poor for feeding (80%, 52%, 55%) and standing (80%, 46%, 58%), which are difficult to differentiate using a leg-mounted sensor. Position data made it possible to differentiate feeding and standing. The D-S evidence fusion method for combining accelerometer data and location data in classification was used to fuse two pieces of basic behavior-related evidence into a single estimation model. With this addition, the sensitivity and precision of the two difficult behaviors increased by approximately 20 percentage points. In conclusion, the classification results indicate that the ensemble classifier effectively recognizes various behavior patterns in dairy cows. However, further work is needed to study the robustness of the feature and model by increasing the number of cows enrolled in the trial.

## Introduction

Behavior is an important indicator of health and well-being in dairy cows. Cows exhibit different behaviors when health problems (e.g., lameness) or physiological changes (e.g., oestrus) occur [1]. Behavior assessment is mainly via a farmer's experience, but focused, long-term detection is difficult, especially in large herds, due to lack of time and labor. Some existing systems based on sensor technology have been developed for automatic behavior analysis [2–4], but these systems usually identify only a few behavior patterns. An accurate, fast, and low-cost method for monitoring behavior patterns as an aid in evaluating bovine health and welfare would be beneficial.

With the advantages of being small in size and light weight, accelerometers have been widely used to monitor human behaviors and activities. Many valuable studies have used accelerometers for distinguishing cow behavior patterns [5–8], making use of a variety of machine learning methods, including support vector machines (SVMs), decision tree algorithms, and the *k*-means clustering algorithm [9]. These methods fall into the general categories of supervised learning and unsupervised learning. However, these machine learning algorithms have disadvantages in the form of excessive memory consumption, poor on-line learning ability, high sensitivity to outlier data, and limited performance of global optimization.

Cow location data are a direct reflection of cow behavior. For example, cows feed only near the troughs. This information improves the accuracy of recognizing cow behavior. Several researchers have analyzed the possibility of tracking cows with different wireless systems. Porto et al. demonstrated that an Ultra Wide Band (UWB) system can locate cows with a mean error of about 0.11 m (with an identification accuracy of nearly 100% for the reference tag) in a semi-open free-stall barn [1]. Huhtala et al. achieved a localization accuracy of 0.1 m in a test using a wireless sensor network (WSN) and the time difference of arrival (TDOA) algorithm [10]. However, neither of these studies used acceleration data.

The integration of accelerometers with real-time location data has the potential to achieve better results for bovine behavior identification. There are additional benefits to incorporating acceleration data. Such monitoring systems can simultaneously identify, locate, and manage dairy cows. Location data augmented by acceleration improves the recognition of bovine behaviors and enables the early detection of health problems in cows.

The main objective of this study is the development and evaluation of a new method to combine acceleration and location data using evidence theory to improve the accuracy of bovine behavior recognition. Evidence theory, also known as D-S evidence theory, is a method of reasoning with uncertainty proposed by Dempster and Shafer in the 1960s [11, 12]. Evidence theory offers multiple inaccurate descriptions of the problem studied and then reaches an inaccurate conclusion by focusing on the consistency in the descriptions according to a specific measure. Evidence theory can be used for both data fusion and pattern recognition [13–15]. Our research has two main purposes. First, we develop a complete design and specific property of devices for continuous surveillance of cow behavior. Second, we execute a series of tests to validate the system performance of automatic detection for various types of cow behavior patterns.

## Materials and methods

### Ethics statement

During our research, all animals were kept in a pathogen-free environment and fed naturally. The procedures for care and use of animals were approved by the Ethics Committee of the Henan University of Science and Technology, Luoyang, China. All of the experimental procedures were conducted in conformity with institutional guidelines for the care and use of laboratory animals at Henan University of Science and Technology and with the National Institutes of Health Guide for Care and Use of Laboratory Animals (NIH Pub. No. 85–23, revised 1996).

### The data acquisition system

**Fig 1:**
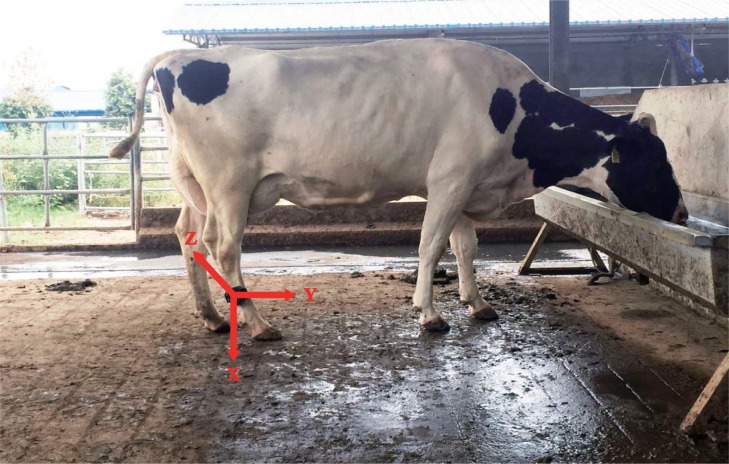
Coordinate system of the leg-tag.

Our system hardware consisted of five leg tags, six location sensors (Sensor 1 through Sensor 6), and one reference location sensor. The leg tag sensor enabled automated measurement of both acceleration and location of cow movements. Each leg tag included a three-dimensional accelerometer (ADXL345, Analog Devices Inc., USA), a radio frequency (RF) transceiver (CC1101, Texas Instruments Inc., USA), and a microcontroller (STC12C5A60S2, STCmicro Technology Inc., China). The accelerometer used to obtain acceleration data has a range of ±8 g and a sampling frequency of 1 Hz. It integrates a 12 bit A/D converter to change the analog voltage signal into digital data. The RF transceiver transmits acceleration and location measurements to a laptop computer once per second at 433 MHz and receives ranging data from location sensors using received signal strength indicator (RSSI) technology to analyze leg tag locations. Each leg tag measures 89 × 60 × 38 mm and is placed in a water-resistant plastic bag. The power supply consists of three 3.7 V lithium ion batteries (ARB-L4-4800, FENIX Ltd., China). The protected tag is inserted into a plastic case equipped with adjustable straps and an adhesive label showing the identification code of the specific leg tag. The adjustable straps enable a proper fit of the leg tag to the dimensions of cow's hind leg in order to have the *y*-axis of the coordinate system of the leg tag aligned with the axis of cow body (Fig 1).

The weight of leg tag circuit, plastic bag, batteries, plastic case, and adjustable straps is approximately 30.8 g, 107.5 g, 121.5 g, 268.2 g, and 71.4 g, respectively. The total weight of each leg tag is approximately 600 g, most of which is from protective and mounting components that ensure reliability. The plastic bag is made of TPU (thermoplastic urethane, 1.25 g/cm^3^), and the plastic case is PLA (polylactic acid, 1.3 g/cm^3^). The density of these materials is high, but lighter weight materials could be considered in the future. We used motion video analysis software (MIAS, Fubo Tech Ltd., China) to measure the stride length, speed, and time of leg swing of normal walking or active walking before and after installing the leg tag. The results showed that there was no obvious difference in these parameters. We observed no restriction or influence of the leg tag on the cows’ activities.

The location sensor measures 75 × 48 × 30 mm, weighs 200 g, and communicates with the leg tags using the 433 MHz radio band. Each location sensor is powered by an AC/DC adapter and attached to a pole or similar mast at a height of 2.5 m using cable ties. Each of the six location sensors sends data packets with its own location and identification number (ID) at 0.5 s intervals. The position of Sensor 1 is defined as the origin of the plane coordinate system in the barn. The graphical user interface (GUI) of the corresponding computer application uses the same coordinate system to visualize the actual movement of cows.

Fixed to a post in the center of the barn, the reference location sensor corrects location signals via spatial correlation between the reference location sensor and the six location sensors. This improves location accuracy of the five leg tags by eliminating common ranging errors.

Our specialized application enables users to perform post-processing of the measurement data acquired from the five leg tags. It executes the ensemble classifier to transform measurements into identified behavior. The main parts of our software are platform management, the service control manager, and the data fusion engine (DFE). The first two manage the system configuration and services, animal characteristics (e.g., age, weight, health status, and picture), the graphical representation of the monitoring area, the wireless communication parameters, the display of data obtained from the leg tags, and the statistical analysis of the recorded data. The third part, the DFE, is a real-time processing algorithm which synthesizes the acceleration and location data collected by the system. Using D-S evidence theory, the DFE enables finding a straightforward solution to improve the classification performance of cow behaviors.

We used a MySQL database to store the acquired information. The application records the latest data from the leg tags at 1 second intervals. The data recorded for each tag includes the acquisition date and time, the identification number, the three-dimensional acceleration, and the two-dimensional location in the plane.

We also used a video recording system using four cameras (SNC-VB640, Sony Corporation, Japan) in the barn to validate the results from our system. Among all of the possible views available from the video system, plan views are the most appropriate here. A top view of the system provides a panoramic image of the area of interest at a resolution of 1920 × 1080 pixels. We compared the behaviors shown in the images with the acceleration data acquired from the sensor system via vision processing software.

We synchronize the video images with the leg tag data in order to match the video analysis with the accelerometer data for each cow. We vary the time of video recording from 9 a.m. to 11 a.m. to obtain video of each cow involved in all activities (feeding, lying, standing, lying down, standing up, normal walking, and active walking). The same person downloads and logs the video recordings and determines the activity for all cows. The following criteria determine the activity classification.

- Feeding: If a cow stands on its four legs at the feeding zone, and lowers its head into the headlocks to search or masticate the feed for the entire 1 s video period, the activity is categorized as feeding.
- Lying: If an animal is lying down for the entire 1 s video period, the activity is classified as lying. When an animal transitions from this position, the lying activity classification ends once the first movement of the transition occurs.
- Standing: Static standing and standing with minor limb movements (shifting) for the entire 1 s video period leads to a classification of standing.
- Lying down: When a cow completes a transition from standing to lying for a 3 s to 8 s video period, the activity is categorized as lying down.
- Standing up: Similar to the lying down activity, if the duration of the conversion from lying to a standing takes place during a 3 s to 8 s video period, the activity is categorized as standing up.
- Normal walking: Normal walking is defined as a progressive step (forward or backward) within a 1 s video period.
- Active walking: Active walking activity is defined as a minimum of two progressive steps (forward) within a 1 s video period.

### Housing and animals

We conducted a trial from 1st June to 20th June 2017 at a dairy farm located in Nanyang (Henan Province, China) to investigate our system’s performance. In the study, the free-stall barn (33°05′50.64″N, 112°32′25.32″E) had a rectangular layout of 180 m × 31 m in an east-west direction and included a feeding passage, two rows of self-locking headlocks, and two rows of head to head stalls arranged with sand beds. The roof was covered with light-weighted color steel plates with symmetric structure with a 1:3 slope. The height of the barn and the eaves was 10 m and 4.65 m, respectively.

**Fig 2:**
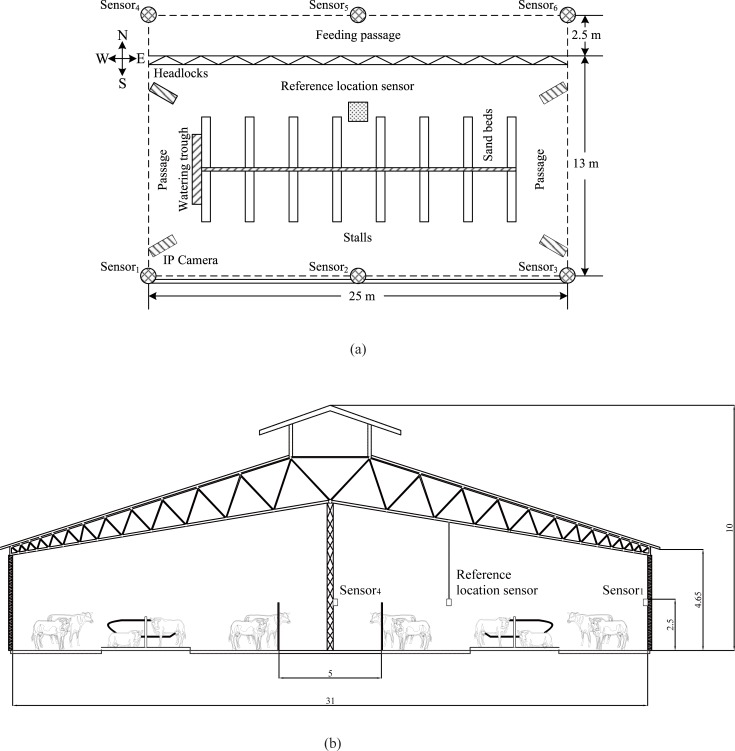
Plan and section of the studied area in the barn. (a) Plan. (b) Section.

The separation area in the barn had 11 cows, five of which were chosen for the trial on the basis of similar body size. All five of the cows were in the early lactation stage (101–117 days in milk). The cows were multiparous with parity in the range of 2 to 3 (3 cows in 2nd lactation, 2 cows in 3rd lactation). The five Holstein dairy cows (designated as ID1, ID2, ID3, ID4, and ID5) were loose-housed in the studied area (Fig 2). The studied area measured 25 m× 13 m and was located in the middle of the barn and separated by fences. The internal facilities included a watering trough, a row of self-locking headlocks, and seven groups of head to head stalls. Cows were milked twice a day, around 5 a.m. and 5 p.m., with a fish-bone milking machine. Floors were cleaned daily at 7 a.m. with a scraper blade. Cows were fed the total mixed ration (TMR) diet to achieve balanced nutrition. No behaviors were forced during the experiment. None of the five cows had shown any signs of serious lameness or other disease that would affect their behavior.

As mentioned previously, we acquired data with both our proposed system and video recorders. The leg tags were sampled once per second (1 Hz), with measurements gathered immediately by the wireless data collection system. Video observation occurred simultaneously according to an ethogram defined in advance for all behavior categories (Table 1). Afterwards, the video and leg tag observations could be aligned with each other on the basis of the timestamps. As noted already, the video recordings were observed manually in order to assign the cow behavior in each time frame to one of the defined categories.

**Table 1:** Descriptions of the predefined behaviors.

Activity level	Behavior category	Definition
Inactive behavior^a^	Feeding	The cow is at the feeding zone and searches for or masticates the feed.
	Lying	The cow is in a cubicle in a lying down position.
	Standing	The cow stands entirely on its four legs.
Active behavior^b^	Lying down	The cow bents one foreleg, lowers its forequarters, then hindquarters, and settles down in a state of lying.
	Standing up	The cow rises from a lying state to stand on all four feet.
	Normal walking	The cow changes its location in space either in forward or backward direction with a minimum of one stride within 1 s.
	Active walking	The cow changes its location in space in a forward direction with a minimum of two strides within 1 s.

^a^ The cow has little or no movement of legs.

^b^ The cow has significant and continuous leg movements.

To save labor during video classification, we developed a simple image analysis program to detect obvious motion in the video to assist the observer. We used a block matching method [16] to detect motion. The matching algorithm is a motion estimation method for locating matching macroblocks in a sequence of digital video frames. It is widely used in target detection and video compression. The block matching algorithm divides the current frame of a video into macroblocks and compares each of the macroblocks with a corresponding block and its adjacent neighbors in the previous frame of the video. The algorithm produces a vector map indicating orientation and strength of the motion. We configured the detection threshold to detect obvious motion of cows. The algorithm divided the video flow into pieces which were then evaluated by human observation and marked as ground truths. The different behavior activities were further extracted from the measurements with the support of the video data. The behaviors of each cow were constantly monitored during a two-hour daily window (9 a.m.–11 a.m.), yielding 200 observed hours in total.

### Cow behavior recognition

#### AdaBoost

We applied the AdaBoost (Adaptive Boosting) algorithm to behavior recognition in this study. Developed by Freund and Schapire, AdaBoost is a classifier ensemble algorithm using a finite number of weak learners [17, 18]. Its advantages include lower memory and computational requirements. A weak learner (e.g., a single level decision tree or simple neural network) is a simple, fast, and easily implemented classifier with classification accuracy only slightly better than a random guess [19]. However, AdaBoost individually trains its weak learners and combines their decisions to determine a final decision. In other words, the powerful pattern classification capacity of AdaBoost algorithm is formed by iteratively combining performances of weak learners to build a strong classifier whose performance is better than any of the individual weak classifiers.

#### Implementation of classifier

We recorded the start and end times of the behavior for each observation, and we used a program to match observation data with acceleration data automatically. Due to accidental network delays and data packet loss, some observations were removed in the initial processing phase. Table 2 presents the composition of the behavior observations stored in the database.

**Table 2:** Composition of behavior observations.

Behavior pattern	Number of observations		Distributions						
	Original^a^	>5 s^b^	< 4 s	4 s	5 s	6 s	7 s	8 s	> 8 s
Feeding	5239	3676	401	535	627	614	524	607	1931
Lying	7857	6398	353	417	689	732	748	636	4282
Standing	4355	3386	37	369	563	452	513	528	1893
Lying down	1172	449	2	207	514	327	103	15	4
Standing up	1322	378	8	173	763	304	58	14	2
Normal walking	4075	2870	267	476	462	739	642	628	861
Active walking	1901	873	167	372	489	517	321	26	9
Total	25921	18030	1235	2549	4107	3685	2909	2454	8982

^a^ Original number of observations in the database

^b^ Number of observations with the duration over 5 s in the database

The length of the time window directly determined the number of data samples used for training and testing. If the length of the time window was too short, the differences between the measurements for each behavior were not obvious or significant, which would directly affect the classification performance of the algorithm. By increasing the time window to 5 s or longer, the measurements effectively contained the whole process of all behavior activities to ensure the integrity of behavior data. Our system included only those observations lasting more than 5 s in the training and performance evaluation of the modeling process. The number of observations with a duration over 5 s in the database was 18030 (Table 2). The number of lying down, standing up and active walking observations decreased dramatically when applying the >5 s filter. We divided the data samples randomly into classifier training and testing data sets. Sixty percent of the data (10818 sets) was selected as the training data set, and the remaining 40% (7212 sets) was used for the testing set. We trained the classifier model with the accelerometer measurements (input) and the corresponding behavior categories (output).

We implemented the AdaBoost algorithm by combining a multi-class BP (Back Propagation) neural network with the Stagewise Additive Modeling using Multi-class Exponential loss function (SAMME) algorithm to construct a strong classifier [20, 21], hereafter called the Multi-BP-AdaBoost algorithm. The AdaBoost algorithm used the back propagation neural network as the weak learner to predict the output of samples via iterative training. We also optimized the number of iterations for the Multi-BP-AdaBoost algorithm. We performed 30 training runs to determine the optimal number of iterations for best classification accuracy. Algorithm 1 summarizes the steps of the Multi-BP-AdaBoost algorithm.

Algorithm 1. Multi-BP-AdaBoost algorithm.

Input: The training set *T* = {(*p*_1_,*q*_1_),⋯,(*p*_*N*_,*q*_*N*_)}, where *p*_*i*_ ∙ (*p*_*i*_ ∈ *P* ⊆ *R*^3^) represents the three-axis acceleration data, *q*_*i*_ refers to the matching behavior class, *P* expresses the training set containing 7 types of cow behaviors, and *N* denotes the total number of samples in the training set.

Output: The AdaBoost classifier *G*(*p*).

1. Initialize the parameters of the Multi-BP-AdaBoost algorithm, including the number of iterations (*L*) and the weight *ω*_1*i*_ of each training sample, where *ω*_1*i*_ is formally expressed as $\omega_{1i}=\frac{1}{N}$, *i* = 1,⋯,*N*.
2. **for** *l* = 1 **to** *L* **do**
3. Train the training set samples to obtain the BP weak classifier as follows: *G*_*l*_(*p*):*P*→{*K* = 1,2,⋯,7}, where *K* is an enumeration of the 7 recognized behaviors.
4. Compute the error rate of classifier: $err_{l}=\sum_{i=1}^{N}\omega_{li}\times I(G_{l}(p_{i})\neq q_{i})$. *I* returns 1 when the condition in parentheses is satisfied and 0 otherwise.
5. Compute the coefficient of *G*_*l*_(*p*): $a_{l}=\frac{1}{2}\log(\frac{1-err_{l}}{err_{l}})+\log(K-1)$.
6. Update the weights of the training samples to be used in the next iteration (*l*+1): $\omega_{l+1,i}=\frac{\omega_{li}}{\sum_{i=1}^{N}\omega_{li}\exp(-a_{l}q_{i}G_{l}(p_{i}))}\exp(-a_{l}q_{i}G_{l}(p_{i}))$, *i* = 1,⋯,*N*.
7. **end for**
8. Output the final classifier: $G(p)=int(\sum_{l=1}^{L}a_{l}G_{l}(p))$. *Int* is applied to return only the integer part of the argument, which then maps to a behavior in set *K*.

### Location information acquisition

**Fig 3:**
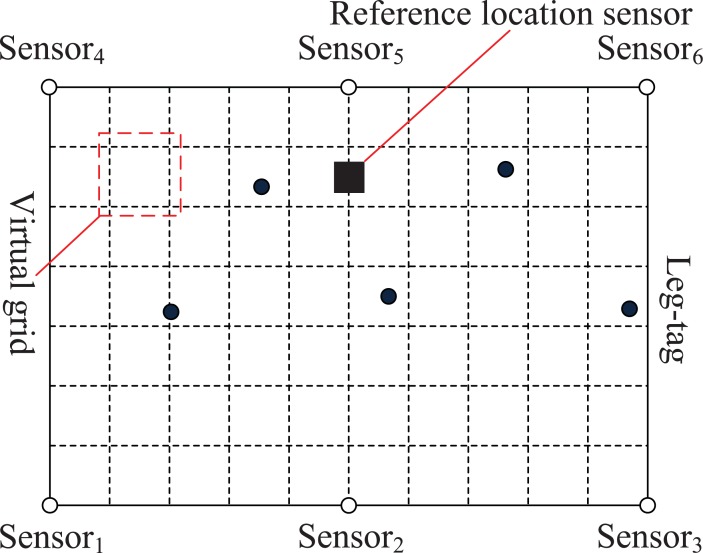
Grid plot of the area of the barn under study.

The location Sensor 1 was defined as the sink node with collected information (locations, IDs, etc.) sent by each of the other location sensors. According to the physical distribution of the location sensors, Sensor 1 divided the studied area in the barn into virtual grids of equal size (Fig 3). Each grid consisted of four vertices and four edges. The vertices in addition to the boundary of the studied area were expressed as *O*_*j*_ (*j* = 1, 2,…, (*S*-1)^2^, *S* represents the number of equal parts of the boundary when the virtual grids are divided). The estimated distance from each leg tag to a given location sensor was defined as *d*_*i*_ (1≤*i*≤6) and obtained by the RSSI value received. The combined distances formed a distance vector *D* (*d*_1_, *d*_2_,…, *d*_6_). It could be testified that there was a nonlinear mapping relationship between location of each leg tag and the corresponding distance vector.

The communication between each leg tag and the location sensors provided the RSSI ranging data, which was gathered into a vector *R* (*r*_1_, *r*_2_,…, *r*_6_) where each element indicates the received signal strength at each location. We translated the vector *R* into the distance vector *D* (*d*_1_, *d*_2_,…, *d*_6_) using the lognormal shadowing model in order to gain a better ranging effect [22]. The distance from one vertex of virtual grids in addition to the boundary of the studied area to the location sensors was defined as *h*_*i*_ (1 ≤ *i* ≤ 6), and then a distance vector *H* was constituted as (*h*_1_, *h*_2_…, *h*_6_). We used a similarity function to calculate the proximity of a leg tag to each vertex:

$$E(D,H)=\sum_{i=1}^{6}\left(1-\frac{\left|d_{i}-h_{i}\right|}{\left|d_{i}-h_{i}\right|+u_{i}}\right)/6,$$ (1)

where *d*_*i*_ is the *i*th entrance of the distance vector *D*, *h*_*i*_ is the *i*th entrance of the distance vector from one of vertices of virtual grids in addition to the boundary of the studied area to all location sensors (the number of *H* was equal to (*S*-1)^2^), and *u*_*i*_ is the absolute average of *d*_*i*_ and *h*_*i*_. After calculating the similarity values of the distance vector *D* with the distance vector *H* for each vertex according to Eq (1), the corresponding array *C* was calculated as the similarity values between a leg tag and all vertices in addition to the boundary of the studied area, which was (*S*-1)^2^ in length. The array *C* was expressed as:

$$C=[E_{1},E_{2},\cdots ,E_{{\left(S-1\right)}^{2}}],$$ (2)

where *E*_*i*_ is the similarity value of the distance vector *D* with the distance vector *H* of the *ith* vertex calculated according to Eq (1).

The coordinates of the leg tag were obtained by computing the centroid of the three vertices with the top three similarity values. The centroid was calculated as:

$$\left(X,Y\right)=\left(\frac{\sum_{j=1}^{3}X_{j}}{3},\frac{\sum_{j=1}^{3}Y_{j}}{3}\right),$$ (3)

where (*X*, *Y*) are the coordinates of the leg tag, and (*X*_*j*_, *Y*_*j*_) are the coordinates of the vertices with the highest similarity.

Environmental factors (e.g., temperature, humidity, air pressure) occasionally affected the wireless signal. Fluctuations in the RSSI measurements were minor. However, these factors did affect the distance estimation of the determined propagation model (i.e., lognormal shadowing model), which resulted in some errors in the RSSI ranging measurements. Accordingly, there were regular errors in the distance measurements between the leg tags and the location sensors in the actual work environment. To improve location accuracy, we corrected the measured distances through the reference location sensor by comparing real distances with measured distances and applying an error correction coefficient. This coefficient is defined according to:

$$\delta =\frac{1}{6}\sum_{i=1}^{6}\frac{e_{i}-f_{i}}{f_{i}}$$ (4)

$$d_{i}^{\prime }=d_{i}(1+\delta ),$$ (5)

where *e*_*i*_ is the real distance from the reference location sensor to the *i*th location sensor, and *f*_*i*_ is the measurement distance from the reference location sensor to the *i*th location sensor based on RSSI. The real distance between the reference location sensor and each location sensor was determined by their position relationship after the system was deployed. The measured distance from the reference location sensor to each location sensor was obtained by converting the RSSI value between each sensor pair into a distance value using the lognormal shadowing model. The *δ* is the error coefficient as defined in Eq (4), which reflects the accuracy of the measured distances from the reference location sensor. The parameter *d*_*i*_ is the measured distance from one leg tag to the *i*th location sensor, and $d_{i}^{\prime }$ is the corrected distance.

### Data fusion method using D-S evidence theory

The position of the leg tag on the body of the cow can affect the performance of classification, as illustrated by Moreau et al. (2009) who deployed sensors at different positions on the body of a goat when classifying grazing behavior [23]. Feeding and standing are the behaviors most likely to be misclassified using a leg-mounted sensor. The hardware used in this study was an effective wireless sensor network that tracked the spatial position of each cow precisely. The addition of acceleration data enabled more accurate identification of biologically-relevant behaviors (e.g., feeding and standing).

No behavioral classification algorithm will ever be free from error, but D-S evidence theory promises better differentiation between feeding and standing by combining multiple indicators into a single model. The data fusion method used two behavior-related features, namely the discriminant result of feeding or standing behavior provided by the Multi-BP-AdaBoost algorithm and the position information of cow, as independent sources of evidence.

The first step in applying D-S evidence theory was defining the propositional space of possible solutions, the frame of discernment, denoted by Θ [24]. The set of all subsets of Θ were denoted by 2^Θ^. In the case of behavior classification, the frame of discernment contained 2 elements, feeding and standing. All possible subsets were {feeding}, {standing}, {feeding, standing} and {*ϕ*}. The subset {feeding, standing} denoted the subset unable to distinguish feeding or standing, which was simplified as {Uncertainty}. {*ϕ*} denoted an empty subset that was not involved in evidence fusion. Consequently, *A* represented all the subsets of Θ, which specifically included {Feeding}, {Standing}, {Uncertainty}, {*ϕ*}. We also defined basic probability assignments (BPAs) in accordance with D-S evidence theory, giving probabilities to each element in 2^Θ^ according to evidence. We expressed BPAs as the mass function *m* indicating the probability of each behavior, where *m*: 2^Θ^→[0, 1], *m* (*ϕ*) = 0, and $\sum_{a\in A}m(a)=m(\mathrm{Feeding})+m(Standing)+m(\mathrm{Uncertainty})+m(\phi )=1$.

Table 3 shows the design of the BPA functions. Assignments were based solely on evidence, allowing the representation of ignorance. Considering the confusion between feeding and standing, the probability of feeding and standing together was regarded as an approximate status. For the evidence of the classification result on feeding or standing, the probability of the behavior estimated by the Multi-BP-AdaBoost algorithm was set to 0.5, the probability of another behavior was set to 0.4, and the probability of uncertainty was set to 0.1. We assumed that when the cow's hind leg was located about 1.5 m away from the headlocks, the cow was most likely in the state of feeding because 1.5 m is the average length of a cow’s body excluding the head. In contrast, when the hind leg was close to 0 m from the headlocks, the cow was likely to be parallel to or facing away from the headlocks and was more likely in the state of standing. If the cow was located away from the headlocks within the studied area, then standing had greater probability.

**Table 3:** Basic probability assignment functions based on interval division.

Evidence	BPAs	Interval division	*m*(Feeding)	*m*(Standing)	*m*(Uncertainty)
Result of Multi-BP-AdaBoost algorithm	*m*_1_	Feeding	0.5	0.4	0.1
		Standing	0.4	0.5	0.1
Position of cow	*m*_2_	0 < *D*^a^ ≤ 1.5 m^b^	$\left(1-\frac{\left(1.5-D\right)}{1.5}\right)\times 0.9$	$\frac{\left(1.5-D\right)}{1.5}\times 0.9$	0.1
		1.5 m < *D* ≤ 1.5 m + *error*_*max*_^c^	$\left(1-\frac{\left(D-1.5\right)}{(1.5+error_{max})-1.5}\right)\times 0.9$	$\frac{\left(D-1.5\right)}{(1.5+error_{max})-1.5}\times 0.9$	0.1
		1.5 m + *error*_*max*_ < *D* ≤ 13 m^d^	0	0.9	0.1

^a^ Perpendicular distance between cow's hind leg and headlocks

^b^ Average length of a cow’s body excluding the head

^c^ Maximum value of mean positioning errors of the cows under study

^d^ Width of the experiment area in this study

We fused the evidence (i.e., behavioral indicators) via combination rules and combined the corresponding BPAs into a single model using Dempster’s rule of combination. For behavior-related features, the BPAs of the two independent indicators were represented by *m*_1_ and *m*_2_ (Table 3). The combination operator in Dempster’s rule, denoted by ⊕, satisfies the commutative and associative laws. We fused conflicting evidence from the two sources using the following relation:

$$\left\{ \begin{array}{l} m^{\prime }=m_{1}⊕m_{2}=(m^{\prime }(\mathrm{Feeding}),m^{\prime }(\mathrm{Standing}),m^{\prime }(\mathrm{Uncertainty}))\\ k_{1}=m_{1}(\mathrm{Feeding})m_{2}(\mathrm{Feeding})+m_{1}(\mathrm{Feeding})m_{2}(\mathrm{Uncertainty})+m_{2}(\mathrm{Feeding})m_{1}(\mathrm{Uncertainty})\\ k_{2}=m_{1}(\mathrm{Standing})m_{2}(\mathrm{Standing})+m_{1}(\mathrm{Standing})m_{2}(\mathrm{Uncertainty})+m_{2}(\mathrm{Standing})m_{1}(\mathrm{Uncertainty})\\ k_{3}=m_{1}(\mathrm{Uncertainty})m_{2}(\mathrm{Uncertainty})\\ K=k_{1}+k_{2}+k_{3}\\ m^{\prime }(\mathrm{Feeding})=k_{1}/K\\ m^{\prime }(\mathrm{Standing})=k_{2}/K\\ m^{\prime }(\mathrm{Uncertainty})=k_{3}/K \end{array}\right.,$$ (6)

where *m*′ is the result of combination, and the components *m*'(Feeding), *m*'(Standing) and *m*'(Uncertainty) are the probabilities of feeding, standing, and uncertainty after evidence fusion, respectively. *K*, *k*_1_, *k*_2_, and *k*_3_ are intermediate variables in the fusion process. *m*_1_(Feeding), *m*_1_(Standing), and *m*_1_(Uncertainty) are the probabilities of feeding, standing, and uncertainty determined by result of the Multi-BP-AdaBoost algorithm, and the *m*_2_(Feeding), *m*_2_(Standing) and *m*_2_(Uncertainty) are the probabilities of feeding, standing, and uncertainty computed from the cow’s position. The criterion of feeding or standing was eventually determined by the rules, as given in Eqs (7) and (8).

$$\mathrm{Feeding}:\left\{ \begin{array}{l} m^{\prime }(\mathrm{Feeding})>\varepsilon_{1}+m^{\prime }(\mathrm{Standing})\\ m^{\prime }(\mathrm{Uncertainty})<\varepsilon_{2}\\ m^{\prime }(\mathrm{Feeding})>m^{\prime }(\mathrm{Uncertainty}) \end{array}\right..$$ (7)

$$\mathrm{Standing}:\left\{ \begin{array}{l} m^{\prime }(\mathrm{Standing})>\varepsilon_{1}+m^{\prime }(\mathrm{Feeding})\\ m^{\prime }(\mathrm{Uncertainty})<\varepsilon_{2}\\ m^{\prime }(\mathrm{Standing})>m^{\prime }(\mathrm{Uncertainty}) \end{array}\right..$$ (8)

In these equations, *ε*_1_ and *ε*_2_ are predefined threshold values. In general, *ε*_1_ is significantly larger than *ε*_2_ to ensure classification reliability. According to experience, *ε*_1_ and *ε*_2_ were set to 0.2 and 0.03, respectively, in our study. If the conditions of Eqs (7) and (8) were not satisfied, the result of behavior classification was considered to be uncertain, with related behavioral data removed from recognition and processing.

### Data analysis

We presented our classification results in the form of a confusion matrix listing the number of cases correctly identified as positive (the modeled behavior) or negative (other behaviors). We labeled misclassifications of negative and positive samples as false positives and false negatives, respectively. We evaluated the performance of the algorithm based on accuracy, sensitivity, and precision, along with their bootstrapped statistics (mean ± S.D.). We calculated these indicators as:

$$\mathrm{Accuracy}=\frac{\left(\mathrm{True}\;\mathrm{Positives}+\mathrm{True}\;\mathrm{Negatives}\right)}{\left(\mathrm{True}\;\mathrm{Positives}+\mathrm{False}\;\mathrm{Positives}+\mathrm{False}\;\mathrm{Negatives}+\mathrm{True}\;\mathrm{Negatives}\right)}.$$ (9)

$$\mathrm{Sensitivity}=\frac{\mathrm{True}\;\mathrm{Positives}}{\left(\mathrm{True}\;\mathrm{Positives}+\mathrm{False}\;\mathrm{Negatives}\right)}.$$ (10)

$$\mathrm{Precision}=\frac{\mathrm{True}\;\mathrm{Positives}}{\left(\mathrm{True}\;\mathrm{Positives}+\mathrm{False}\;\mathrm{Positives}\right)}.$$ (11)

**Fig 4:**
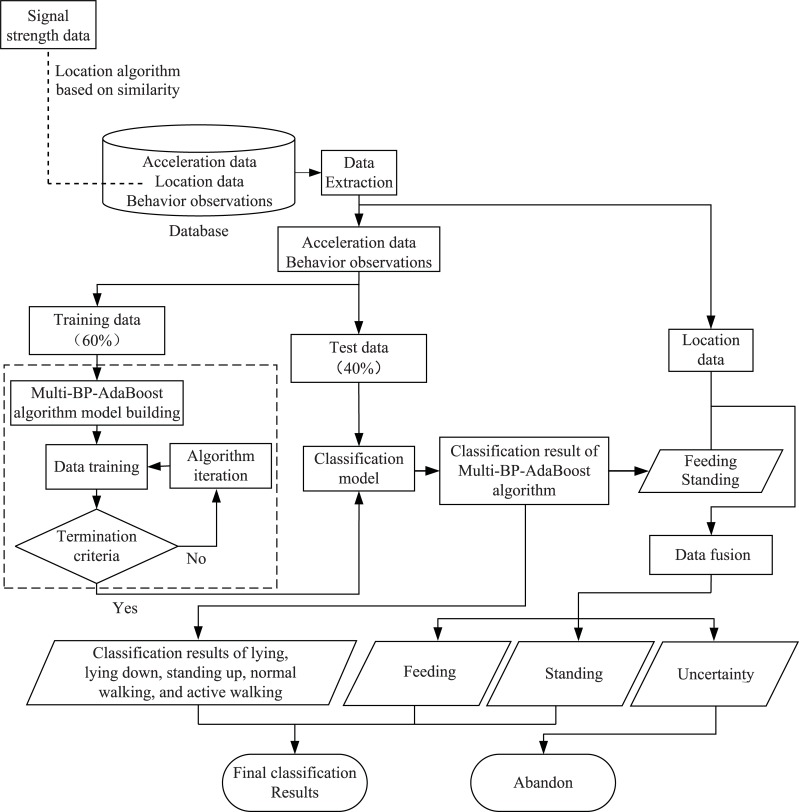
Flowchart of the ensemble classification method proposed in the study.

For each leg tag, we calculated the planimetric positioning error by computing the Euclidean distance between the location provided by the system and that verified by the operator. We used the maximum, mean, and minimum values of the positioning errors to assess the location performance of each leg tag. Fig 4 outlines the ensemble classification method in the study.

## Results

Table 4 shows that the Multi-BP-AdaBoost algorithm clearly identified the specific behaviors of lying, lying down, standing up, normal walking, and active walking but struggled with behavior patterns (feeding and standing) with nearly identical profiles. The algorithm confused feeding and standing (in total, 38.0% and 38.2% of the cases, respectively). However, as shown in Table 5, the overall performance of the Multi-BP-AdaBoost algorithm was quite acceptable. Accuracy was high for all activities. Sensitivity was good for all classes of behavior except for feeding and standing. The highest precision was obtained with active walking. Precision for the feeding and standing classifications was considerably lower than the average level.

**Table 4:** Confusion matrix achieved from the classification of dairy cow behaviors by the Multi-BP-AdaBoost algorithm (the number of correctly classified samples is expressed in boldface).

Predicted behavior	Observed behavior							Total^b^
	Feeding	Lying	Standing	Lying down	Standing up	Normal walking	Active walking	
Feeding	**809**	157	517	2	0	76	0	1561
Lying	60	**2098**	38	1	1	15	2	2215
Standing	559	295	**785**	0	1	44	1	1685
Lying down	4	5	0	**155**	9	6	8	187
Standing up	1	3	1	13	**128**	17	11	174
Normal walking	38	1	13	7	2	**987**	16	1064
Active walking	0	0	0	2	10	3	**311**	326
Total^a^	1471	2559	1354	180	151	1148	349	7212

^a^ Total number of test samples used in the classification

^b^ Total number of behaviors predicted by the Multi-BP-AdaBoost algorithm for each behavior pattern

**Table 5:** Summary statistics (mean±S.D.) of the performance indicators of the Multi-BP-AdaBoost algorithm for all behavior categories.

Behavior pattern	Algorithm performance indicators		
	Accuracy^a^	Sensitivity^b^	Precision^c^
Feeding	0.80±0.03	0.52±0.02	0.55±0.01
Lying	0.92±0.01	0.93±0.01	0.82±0.02
Standing	0.80±0.04	0.46±0.01	0.58±0.01
Lying down	0.99±0.02	0.82±0.01	0.86±0.01
Standing up	0.99±0.00	0.74±0.04	0.85±0.01
Normal walking	0.97±0.04	0.92±0.01	0.86±0.03
Active walking	0.99±0.02	0.94±0.01	0.89±0.02

^a^ Proportion of predictions (positive or negative) that were correct

^b^ Proportion of positive predictions that were correct

^c^ Proportion of the positive cases that were predicted positive

During the selected experiment time, we verified cow positions using rectified panoramic top-view images from the video-recording system. We created the dataset of true cow positions by applying our custom processing software that made a direct comparison between location data acquired from the location algorithm with the video based on similarity.

The location performance of the system was better under static conditions than in the case of moving states. Table 6 shows the maximum, mean, and minimum values of the planimetric positioning errors for each of the five leg tags and the reference location sensor. The positioning error of the system for the reference location sensor was considerably lower than the errors computed for the moving leg tags (Table 6). This was because the position of the reference location sensor was fixed during the trial.

**Table 6:** Location performance of the algorithm based on RSSI similarity degree with each location sensor.

Location sensor	Location performance indicators		
	Maximum value (m)	Mean value (m)	Minimum value (m)
Leg tag_1_	1.37	1.04	0.83
Leg tag_2_	1.24	1.16	0.87
Leg tag_3_	1.26	1.15	0.92
Leg tag_4_	1.31	1.13	0.72
Leg tag_5_	1.41	1.30	1.09
Reference location sensor	0.96	0.85	0.52

After applying the localization algorithm based on the RSSI similarity degree, the resulting average of mean positioning errors of the five leg tags was approximately 1.16 m. The worst mean positioning error (leg tag_5_) of 1.30 m is lower than the average length of a cow’s body excluding the head, which confirmed that location information could be further used for discriminating between feeding and standing. This error would not have affected the analysis of corresponding behavioral indices that did not require a high level of location precision. Moreover, the reference location sensor had higher positioning accuracy and could correct the range errors of the leg tags through Eqs (4) and (5).

The data samples predicted as feeding or standing at the first stage were re-analyzed through the data fusion method using D-S evidence theory (Table 7), with the Multi-BP-AdaBoost classification result and the cow’s position as input. Table 7 shows the re-classification results with significant improvements recognizing the two behaviors. We concluded that combination of indicators is a recommended classification method for behaviors with no obvious differences. However, the inability to satisfy the conditions of classification criterion meant that 77 data samples were considered to be uncertain and abandoned. Table 8 summarizes the performance of data fusion method. The sensitivity and precision of recognizing the two behaviors increased by an average of 20 and 18.5 percentage points, respectively. Furthermore, the proportion of true negatives was lower in the re-classification sample and lead to an inevitable decrease in the accuracy of predicting feeding and standing.

**Table 7:** Re-classification results for all the data samples that have been predicted as feeding or standing using D-S evidence theory (The number of correctly classified samples is denoted in boldface).

Predicted behavior	Observed behavior		Total^b^
	Feeding	Standing	
Feeding	**1178**	435	1613
Standing	347	**1209**	1556
Uncertainty	36	41	77
Total^a^	1561	1685	3246

^a^ Total number of data samples used in the re-classification

^b^ Total number of behaviors predicted by the data fusion method based on D-S evidence theory for feeding, standing and uncertainty

**Table 8:** Summary statistics (mean±S.D.) of the performance indicators of the data fusion method for feeding and standing.

Behavior pattern	Algorithm performance indicators		
	Accuracy	Sensitivity	Precision
Feeding	0.75±0.03	0.73±0.03	0.75±0.04
Standing	0.75±0.04	0.78±0.04	0.72±0.03

## Discussions

Behavior is now recognized as an essential indicator of bovine health that can safeguard farmers from economic distress. However, traditional observational techniques often fail to provide the necessary level of diagnostic accuracy because of time and labor. Accelerometers have been shown to be a feasible method for an automated, remote measurement of behavior patterns in cows [25, 26]. Farmers can benefit from alerts generated from the combination of sensor and accelerometer data. The classification accuracy of the leg tags designed in this study directly influences the utility of this technique. Continuous video observation of cow activities, as implemented in this trial, is the reference standard for evaluating sensor-based measurement. The video-recording system enables accurate recording of both patterns (specific behaviors) and intensity (number, duration, and frequency). Several studies of dairy cow behavior have used video validation [27, 28].

In this study, we developed a Multi-BP-AdaBoost classification algorithm that uses accelerometer data from a leg-mounted sensor to distinguish behaviors. Our evaluation of the algorithm was based on accuracy, sensitivity, and precision.

Our prediction method appears capable of detecting behavior changes using accelerometer data. The performance of Multi-BP-AdaBoost algorithm as presented in the Table 5, was higher than the previous findings of Martiskainen et al. (2009) with the exceptions of feeding and standing [9]. This was reasonable due to the placement of the accelerometer on the leg of the cow, which made distinguishing feeding and standing more difficult as compared to a neck-mounted tag. Therefore, we applied a mixed classification method to strengthen the identification of easily-confused behaviors. In addition, our data had fewer samples of “standing” behavior, which lowered sensitivity of predicting this behavior considerably as compared to other behavior categories.

Since the Multi-BP-AdaBoost algorithm did not reliably differentiate feeding and standing based on the leg tag alone, we introduced the cow’s location. The Multi-BP-AdaBoost algorithm result and the cow’s position were used as two independent indicators to classify behaviors identified as feeding or standing in the first stage. By applying both accelerometer data and location data provided by the leg tag, the algorithm based on D-S evidence theory enhanced the classifier performance of the system by combining evidence correlation of behaviors and spatial positions. We determined each cow's position by analyzing signals exchanged between a corresponding leg tag and the six location sensors. We used RSSI, the ratio between received signal power and a reference power, to estimate distances [29]. In our study, the location method using a similarity function had lower accuracy than other complex positioning methods (such as UWB technology) but offered simplicity, lower cost, and lower computational complexity [30]. In our experimental conditions, the results showed that a higher positioning error was achieved for the leg tags applied to the cows than for the reference location sensor (Table 6). This error was always less than 1.5 m, which is a key threshold for differentiating feeding versus standing. 1.5 m was used to divide intervals of BPA functions of feeding and standing for the position information of cow. The placement of the leg tag on the cow's leg made it impossible to accurately distinguish the orientation of the cow from the position information, which may lead to the behavioral misclassification that was also validated in this trial. Furthermore, the small positioning error shows that the system developed in this study has the capability to provide a detailed analysis of the utilization level of different functional areas of the barn for cow activities.

In this work, we focused on the spatial characteristics of behaviors detected in the leg tags, and estimated the confusing behaviors (feeding and standing) using D-S evidence theory. Two basic behavior-related features of dairy cows were used as independent sources of evidence, namely, the classification result of the Multi-BP-AdaBoost algorithm and the position of the cow. By combining these indicators in a complementary way, we built a single model to fuse various opinions. The classification result of the Multi-BP-AdaBoost algorithm was treated for the two circumstances of feeding and standing. Moreover, the position of the cow was divided into three areas according to the distance between cow and headlocks, which exactly reflected the influence of positioning errors on the classification result. The re-classification results show that the method could be useful in identifying feeding and standing with leg-mounted tags (Table 8).

## Conclusion

In this study, our ensemble classifier achieved ideal classification performance. The results of the study showed that the system is a potential solution to behavior classification in dairy cows. The Multi-BP-AdaBoost algorithm can be used to recognize five categories of behaviors apart from feeding and standing. Our proposed D-S evidence fusion method distinguishes these two confused behaviors and has great potential to monitor position-related cow activities in real-time. By increasing the number of enrolled cows, further work is needed to improve the parameters used in the classifier in order to improve the classification accuracy, sensitivity, and precision. The next step in the development of this system is to test large-scale deployment. Once the functionality and reliability has been confirmed on a larger scale, commercialization is possible.

## Supporting information

S1 Table The data sample of cow behaviors in the time window of 6 s. (XLSX)
